# Supplementary material for: Expression of GP88 (Progranulin) Protein Is an Independent Prognostic Factor in Prostate Cancer Patients
Source: Cancers (Basel). 2019 Dec 16;11(12):2029. doi: 10.3390/cancers11122029 (PMC6966571; doi:10.3390/cancers11122029)
Supplement: Supplementary file 1 [file cancers-11-02029-s001.pdf]

cancers

MDPI

Supplementary material

Suppl. Table 1: IRS values for GP88 staining (average of spots/samples per patient).

IRS	N (patients)
0	187
1.30	1
1.50	41
2.00	26
2.30	5
2.50	1
3.00	37
3.50	11
4.00	46
4.50	2
5.00	1
5.30	1
5.50	5
5.60	1
6.00	26
7.00	1
7.50	3
8.00	24
9.00	5
10.00	2
12.00	12
All	442

GP88 staining could be analyzed for 611 spots/samples for 446 PCa patients. The number of spots/samples per patient on the TMA was 1-3, i.e., 177 patients with one spot/sample, 240 patients with two spots/samples and 18 patients with 3 spots/samples per tumor.

cancers

MDPI

Suppl. Table 2: Spearman’s correlation between GP88-/CK20 staining and clinico-pathological data.

		Age at diagnosis	Gleason Score at prostatectomy	Tumor stage	PSA at prostatectomy	CK20 IRS	Followup time OS, DSS	Followup time RFS
**GP88 IRS**	Correlation coefficient	0.008	-0.006	-0.011	**.201**	**.310**	**-.151**	**-.149**
	Significance (2-sided)	0.867	0.91	0.816	**< 0.001**	**< 0.001**	**0.001**	**0.002**
	N	442	412	442	374	442	442	
**Age at diagnosis**	Correlation coefficient		0.055	0.031	0.01	0.006	-0.04	-0.025
	Significance (2-sided)		0.262	0.521	0.85	0.899	0.398	,605
	N		412	442	374	442	442	442
**Gleason Score at prostatectomy**	Correlation coefficient			**.392**	-0.003	0.016	-0.03	-.109
	Significance (2-sided)			**< 0.001**	0.956	0.747	0.545	0.027
	N			412	356	412	412	412
**Tumor stage**	Correlation coefficient				0.018	0.007	**.127**	,067
	Significance (2-sided)				0.732	0.882	**0.008**	.161
	N				374	442	442	442
**PSA at prostatectomy**	Correlation coefficient					**.179**	**-.218**	**-.215**
	Significance (2-sided)					**0.001**	**< 0.001**	**<0.001**
	N					374	374	374
**CK20 IRS**	Correlation coefficient						-.077	-0.73
	Significance (2-sided)						.108	.125
	N						442	442
**Followup time OS, DSS**	Correlation coefficient							**.933**
	Significance (2-sided)							**<0.001**
	N							442

After Bonferroni correction the α-level of significance is at α=0.00833.

cancers

MDPI

Significant values are marked in bold face

Suppl. Table 3A Cross table: GP88 IRS in groups and PSA values in groups.

			PSA in groups		N	P-value
			**<4 ng**	**≥ 4 ng**		
**GP88**	**IRS<2**	**N**	120	80	200	
	**IRS≥2**	N	74	100	174	
**All**		N	194	180	374	**0.001**

Significant value is marked in bold face.

Suppl. Table 3B Cross table: GP88 IRS in groups and CK20 IRS in groups.

			CK20 _IRS in groups		N	P-value
			**IRS<2**	**IRS≥2**		
**GP88**	**IRS<2**	**N**	194	39	233	
	**IRS≥2**	N	120	89	209	
**All**		N	314	128	442	**0.001**

Significant value is marked in bold face.

cancers

MDPI

Suppl. Tab. 4 Association of GP88/CK20 staining with mean OS and mean DSS.

			Kaplan-Meier analysis			
		**N**	**OS**		**DSS**	
			Months	P	Months	P
				**0.005**		**0.015**
**GP88-/CK20-**	Group 0	194	186.8		216.1	
**GP88-/CK20+**	Group 1	39	217.4		237.5	
**GP88+/CK20-**	Group 2	120	162.3		197.5	
**GP88+/CK20+**	Group 3	89	168.5		208.4	

Group 1 is the reference group. The P-value is calculated over all groups. Significant values are in bold face.

Suppl. Table 5 Univariate and Multivariate Cox's regression analyses: Association of GP88/CK20 staining with OS and DSS.

		Univariate Cox's regression analysis						Multivariate Cox's regression ana		
		**N**	**OS**		**DSS**		**N**	**OS**		**DSS**
			HR (95% CI)	P	HR (95% CI)	P		HR (95% CI)	P	HR (95% C
**20-**	Group 0	194	2.5 (0.78-8.26)	0.119	2.0 (0.3-15.8)	0.501	182	3.4 (0.9-14.2)	0.092	2.0 (0.2-16.
**20+**	Group 1	39	reference		reference		37	reference		reference
**20-**	Group 2	120	4.9 (1.5-16.1)	**0.008**	6.3 (0.8-48.6)	0.077	114	6.0 (1.4-25.4)	**0.014**	5.5 (0.7-42.
**20+**	Group 3	89	3.7 (1.1-12.5)	**0.035**	2.5 (0.3-22.2)	0.421	79	4.5 (1.0-19.5)	**0.045**	2.8 (0.3-25.

Significant values are marked in bold face.

Supplemental Figures

A OS

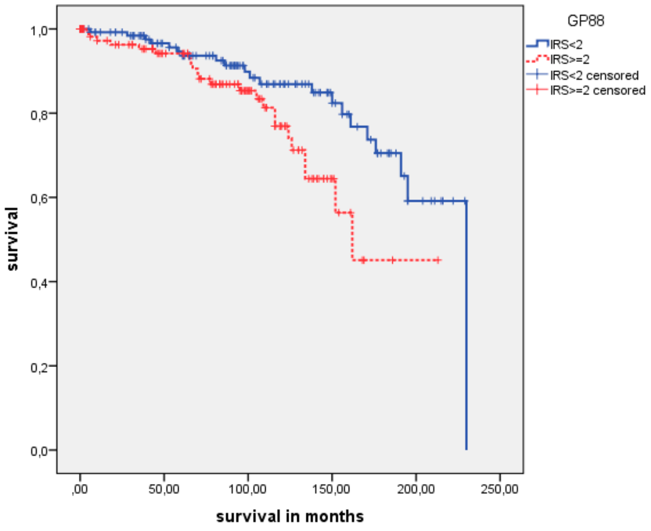

B DSS

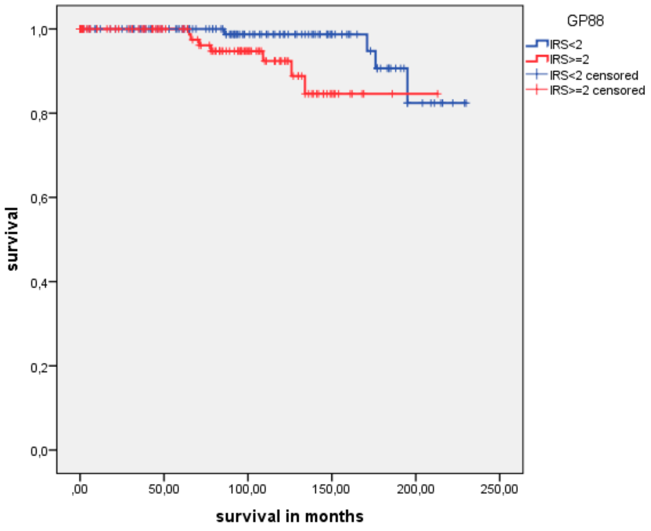

Number at risk					
GP88 IRS<2	137	100	62	33	8
GP88 IRS≥2	123	83	47	9	1

Number at risk					
GP88 IRS<2	137	100	62	33	8
GP88 IRS≥2	123	83	47	9	1

Suppl. Fig. 1 Kaplan-Meier analyses: Association of GP88 staining with prognosis in tumor stage 2 PCa patients

GP88 protein expression was associated with (A) OS (P=0.020) and (B) DSS (P=0.021; all log rank test)

A OS B DSS C RFS

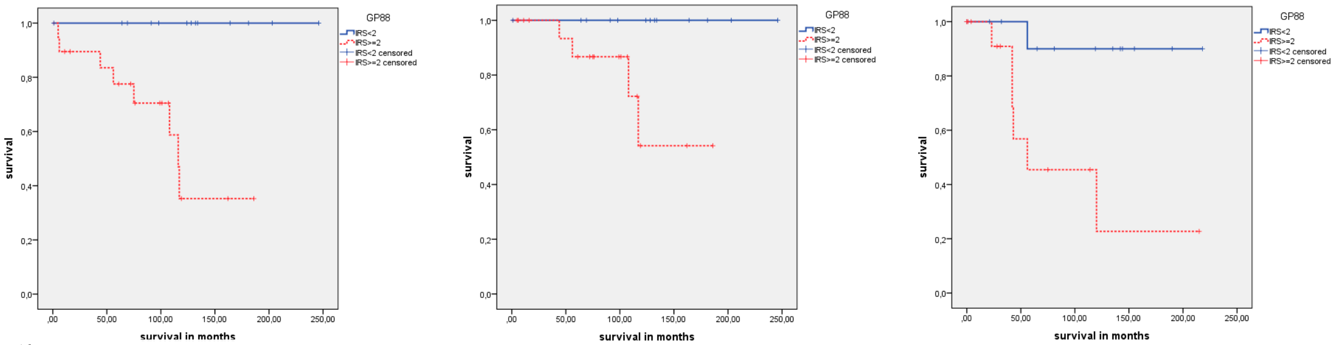

A – Number at risk

GP88 IRS<2	13	12	8	4	2
GP88 IRS≥2	19	14	8	2	0

B – Number at risk

GP88 IRS<2	13	12	8	4	2
GP88 IRS≥2	19	14	8	2	0

C – Number at risk

GP88 IRS<2	13	10	7	3	1
GP88 IRS≥2	14	5	3	1	1

Suppl. Fig. 2 Kaplan-Meier analyses: Association of GP88 staining with prognosis in GS7b (GS8*) PCa patients

GP88 protein expression was associated with (A) OS (P0.004), (B) DSS (P=0.029) and (C) RFS* (P=0.008, all log rank tests).

* GS8* – Gleason Score 8 group for RFS (Gleason score 7b group for OS and DSS)

A OS B DSS C RFS

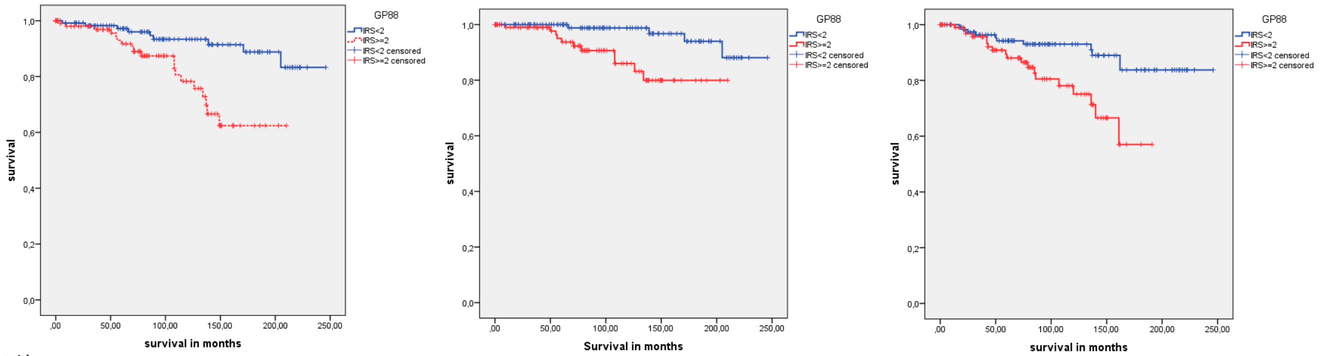

A – Number at risk

GP88 IRS<2	123	94	62	40	17
GP88 IRS≥2	107	75	41	12	2

B – Number at risk

GP88 IRS<2	123	94	62	40	17
GP88 IRS≥2	107	75	41	12	2

C – Number at risk

GP88 IRS<2	123	89	57	37	16
GP88 IRS≥2	107	68	33	9	0

Suppl. Fig. 3 Kaplan-Meier analyses: Association of GP88 staining with prognosis in younger PCa patients (≤65 years)

GP88 protein expression was associated with (A) OS (P=0.001), (B) DSS (P=0.003) and (C) RFS (P=0.004; all log rank tests)

A OS B DSS

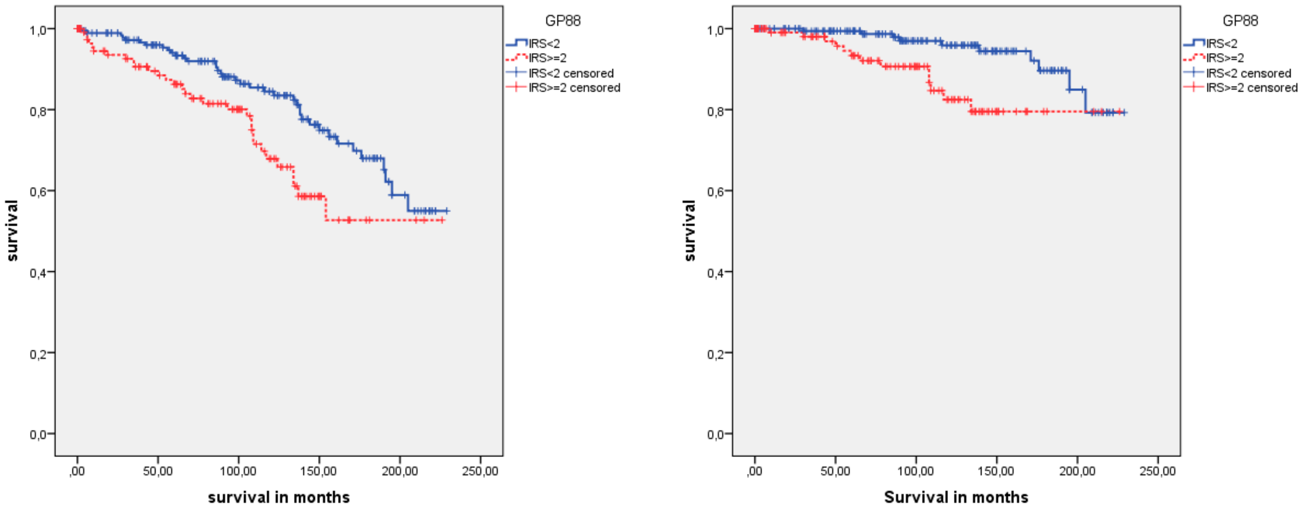

Number at risk

GP88 IRS<2	194	149	100	51	16
GP88 IRS≥2	120	82	51	12	3

Number at risk

GP88 IRS<2	194	149	100	51	16
GP88 IRS≥2	120	82	51	12	3

Suppl. Fig. 4 Kaplan Meier analysis: Association of GP88 staining with prognosis in CK20 negative PCa patients

GP88 protein expression was associated with (A) OS (P=0.009), (B) DSS (P=0.005; all log rank tests)
